# Argon Mediates Anti-Apoptotic Signaling and Neuroprotection via Inhibition of Toll-Like Receptor 2 and 4

**DOI:** 10.1371/journal.pone.0143887

**Published:** 2015-12-01

**Authors:** Felix Ulbrich, Kai Kaufmann, Martin Roesslein, Franziska Wellner, Volker Auwärter, Jürgen Kempf, Torsten Loop, Hartmut Buerkle, Ulrich Goebel

**Affiliations:** 1 Department of Anesthesiology and Intensive Care Medicine, University Medical Center, Freiburg, Germany; 2 Institute of Forensic Medicine, Forensic Toxicology, University of Freiburg, Freiburg, Germany; IISER-TVM, INDIA

## Abstract

**Purpose:**

Recently, the noble gas argon attracted significant attention due to its neuroprotective properties. However, the underlying molecular mechanism is still poorly understood. There is growing evidence that the extracellular regulated kinase 1/2 (ERK1/2) is involved in Argon´s protective effect. We hypothesized that argon mediates its protective effects via the upstream located toll-like receptors (TLRs) 2 and 4.

**Methods:**

Apoptosis in a human neuroblastoma cell line (SH-SY5Y) was induced using rotenone. Argon treatment was performed after induction of apoptosis with different concentrations (25, 50 and 75 Vol% in oxygen 21 Vol%, carbon dioxide and nitrogen) for 2 or 4 hours respectively. Apoptosis was analyzed using flow cytometry (annexin-V (AV)/propidiumiodide (PI)) staining, caspase-3 activity and caspase cleavage. TLR density on the cells’ surface was analyzed using FACS and immunohistochemistry. Inhibition of TLR signaling and extracellular regulated kinase 1/2 (ERK1/2) were assessed by western blot, activity assays and FACS analysis.

**Results:**

Argon 75 Vol% treatment abolished rotenone-induced apoptosis. This effect was attenuated dose- and time-dependently. Argon treatment was accompanied with a significant reduction of TLR2 and TLR4 receptor density and protein expression. Moreover, argon mediated increase in ERK1/2 phosphorylation was attenuated after inhibition of TLR signaling. ERK1/2 and TLR signaling inhibitors abolished the anti-apoptotic and cytoprotective effects of argon. Immunohistochemistry results strengthened these findings.

**Conclusion:**

These findings suggest that argon-mediated anti-apoptotic and neuroprotective effects are mediated via inhibition of TLR2 and TLR4.

## Introduction

Central nervous system injuries such as traumatic brain injury or stroke are one of the leading causes of mortality worldwide [1]. Survival is frequently associated with sustained neurological deficiencies [2, 3]. Generally, neurons are highly sensitive regarding insufficient blood flow or oxygen supply following brain injury. Consequently, nutrient deprivation has an impact upon a multitude of molecular and cellular mechanisms activating apoptotic pathways. This deleterious process is known to end in neuronal cell death.

Neuroprotective drugs aim to reduce secondary brain injury by inhibiting crucial cascades. As a consequence the loss of neurological structures is attenuated and the penumbra is preserved, thus improving recovery [4].

Argon-mediated neuroprotection received increasing attention because of its obvious lack of toxicity and low-cost availability, thus promoting this gas as a promising therapeutic option. Moreover, the absence of anaesthetic activity may be advantageous because argon could be administered to patients without interfering with their actual neurological status.

Recently, we were able to show that argon protects neuronal organs dose- and time dependently and that this effect may be mediated via an ERK1/2 and NF-κB dependent pathway *in-vivo* [5, 6]. Although there have been other numerous investigations aiming to analyse specific pathways (i.e. analysis of GABA receptors, NMDA-receptors, potassium channels [TREK-1] or blocking the K_ATP_-channel)–all of which were possible target of interaction with argon–no effects regarding cytoprotection could be measured [7–10].

So the question remains: How does a gaseous molecule like argon–potentially inert in biological systems–contribute to cellular protection or even to the initiation of specific molecular and intracellular pathway modifications, finally affecting the cells´ fate?

The upstream pathway of our previously demonstrated argon-mediated NF-κB and ERK-1/2 involvement are (among others) toll-like receptors (TLRs), which are signaling receptors of the innate immune system. TLRs play an important role in the processes that lead to and maintain central nervous system injuries [11–13]. By this fact it seems reasonable to hypothesize that argon exerts its anti-apoptotic and neuroprotective effects via TLR signaling.

## Materials and Methods

### Reagents

The TLR4 signaling inhibitor CLI-095 (#tlrl-cli95, TAK-242), and the TLR2+4 inhibitor OxPAPC (#tlrl-oxpap1) were purchased from Invivogen (San Diego, USA). ERK 1/2 inhibitor FR180204 (#SML0320), rotenone, dimethylsulfoxid (DMSO), ionomycin and PMA were obtained from Sigma-Aldrich.

Rotenone was freshly prepared and dissolved in DMSO prior to the experiments. DMSO concentration in cell culture media did not exceed 0.5%. Argon was purchased in fixed gas mixtures (argon 25, 50 or 75 Vol%, oxygen 21%, respective rest nitrogen) from Air Liquide (Kornwestheim, Germany).

### Cell culture and treatment

Neuroblastoma cells (cell line SH-SY5Y; ATCC No. CRL-2266) were grown in DMEM/F12 medium (GIBCO Life Technologies, Darmstadt, Germany)–supplemented with 1% penicillin/streptomycin and 10% fetal calf serum–in a humidified atmosphere with 5% carbon dioxide at 37°C constant temperature until 80% confluence was achieved. The cells were seeded in 6 well culture plates at a density of approximately 1.5 x 10^5^ per well 48 h prior to individual treatment. Prior to rotenone treatment, cells were transferred into media containing 1% fetal calf serum, to prevent inactivation of rotenone by protein binding. Immediately after 4 h of rotenone-treatment, cells were either harvested for further processing or exposed to gas mixtures containing argon 25, 50 and 75 Vol% (oxygen 21 Vol%, carbon dioxide and nitrogen accordingly) for 2 h or 4 h in an air-sealed chamber (dimension of chamber: 38*34*8 cm) in a humidified atmosphere. The initial high flow rate of 8 l/min was reduced to 2 l/min after 5 minutes. During fumigation the temperature was maintained at 37°C.

The inhibitors (TAK-242, OxPAPC and FR180204) were added 60 min. prior to argon treatment. Cells were collected immediately after argon treatment for FACS analysis and quantification or expression of proteins.

### Gas chromatographic analysis of argon concentration

To measure the concentration of argon in the cell culture medium, headspace gas chromatography-mass spectrometry was used (Agilent GC-MS 5793/6890N, Waldbronn, Germany) equipped with a CTC CombiPal Autosampler (CTC Analytics AG, Zwingen, Switzerland). Argon (m/z 40) was detected in full scan mode after separation from other air constituents on a PoraPLOT Q 25x0.25 column (Agilent, Waldbronn, Germany). The method was previously described in detail [14].

Calibration was performed by adding 10, 20, 30, 40, 50, 70 and 80 μL of argon 5.0 (Air Liquide Deutschland GmbH, Düsseldorf, Germany) to blank cell culture medium with a gas tight syringe. Cell culture medium and calibrators were stored at room temperature for 2.5 h prior to analysis to reach equilibrium.

### Flow cytometry of Annexin-V/PI and TLR2 and TLR4

Cells were washed in cold phosphate-buffered saline, trypsinized and resuspended in 100 μl binding buffer (0.01 M HEPES, 0.14 M NaCl, 2.5 mM CaCl_2_, pH 7.4). Staining was performed with 5 μl FITC annexin-V and propidiumiodide (Becton Dickinson, Heidelberg, Germany). Staining for TLR2 and TLR4 was performed using FITC conjugated Anti-TLR4 antibody (Abcam, #ab45126; 1:40) and Anti-TLR2 antibody (Abcam, #ab114070; 1:20). Samples were incubated at room temperature for 15 min before 400 μl of binding buffer was added. Flow-cytometric analysis was done using an acoustic focusing cytometer (Attune^®^, Life Technologies, Darmstadt, Germany). Unstained and single stained cells served as negative controls for background fluorescence and for the set up of compensation and quadrants.

### Western blot analysis

Western blot was performed as previously described [6]. Briefly, equal amounts of protein cell extract (30 μg) were boiled in 5xSDS loading dye (50% glycerol, 0.5 M dithiothreitol, 350 mM SDS, 7.5 mM bromphenolblue, 250 mM TRIS, pH 6.8) for 5 min and subjected to 8%, 10% or 13% sodium-dodecyl-sulfate-polyacrylamide gel electrophoresis. After protein transfer onto PVDF membranes (Immobilon-P, Millipore, Schwalbach, Germany) the membranes were blocked with 5% skim milk in Tween20/PBS and incubated in the recommended dilution of protein specific antibody (p-ERK1/2 #4370, p-IRAK4 #11972 and MyD88 #4283; all Cell Signaling Technology, Danvers, MA, USA, TLR2 #ab24192, TLR4 #ab13556, both Abcam plc, Cambridge, UK) overnight at 4°C. After incubation with a horseradish peroxidase-conjugated anti-rabbit secondary antibody (GE Healthcare, Freiburg, Germany), proteins were visualized using ECL Western blotting detection reagent (Western Lightning plus ECL, #NEL103001EA, PerkinElmer, Waltham, MA, USA) following the manufacturer´s instructions. Images were generated with Fusion Fx^®^ imaging system (PEQLAB Biotechnologie GmbH, Erlangen, Germany). For normalization, blots were re-probed with total ERK1/2 (#4695), total IRAK4 (#4363) and ß-Actin (#4967), all Cell Signaling Technology, Danvers, MA, USA.

### Fluorogenic caspase activity assay

Cells were rinsed with ice-cold phosphate-buffered saline and subsequently cell lysis buffer was added (#70108, Cell Signaling Technology, Danvers, MA, USA). Cells were scraped off the plate and collected in a tube. Cell lysates were diluted to a concentration of approximately 3 mg/ml. Caspase activity assay (Caspase-3 Activity Assay Kit #5723, Cell Signaling Technology, Danvers, MA, USA) was performed according the manufacturer’s instructions. Fluorescence was measured with excitation wavelength at 380 nm and an emission wavelength of 460 nm and expressed in relative fluorescence units [RFU].

### Immunohistochemical staining

Cells were grown to approximately 50% confluency, afterwards using Dako EnVision^TM^ G/2 Doublestain System (Rabbit/Mouse; DAB+/Permanent Red, #K5361) to visualize TLR2 (Abcam, #16894, brown color) and TLR4 (Abcam, #ab13556, red color) expression following different treatments. The assay was performed according the manufacturer’s instructions. Thereafter, cells were stained with hematoxilin for background visualization. Following embedding, images were taken using the Axio-Vision Software (Zeiss, Oberkochen, Germany).

Quantification of immunohistochemistry was done using the ImageJ software (Ver. 1.43u, NIH, USA) and the histogram tool. Photographs of 5 individual experiments (10 fields of vision for each experiment) were taken and the histogram was analyzed in a blinded fashion to the observer.

### Statistical analysis

Data was analyzed using a computerized statistical program (SigmaPlot Version 11.0, Systat Software Inc., San Jose, CA, USA). The results are presented as means (±SD) after normal distribution of data had been verified. Data with normal distribution were compared using one-way ANOVA (α = 0.05) for between-group comparisons with post hoc HolmSidak test. Non-parametric Kruskal Wallis one-way ANOVA on ranks with post hoc Newman-Keuls test was used for data with lack of normal distribution. Kruskal Wallis ANOVA on ranks with post hoc Newman-Keuls test was used to analyze caspase activity assay. *P*<0.05 was considered statistically significant.

## Results

### Concentration of argon in the cell culture medium

To confirm adequate exposure of neuronal cells to argon in our *in vitro* gas application system, we used headspace gas chromatography-mass spectrometry to determine the concentration in the cell culture medium after 2 and after 4 hours of exposure to argon 25, 50 and 75 Vol% (all n = 8). The preceding calibration showed good linearity (R^2^ > 99%). At 2 hours, the concentration of argon 25 Vol% (Table 1) was 19.1±3.2 μL/mL, 50 Vol% 24.9±1.4 μL/mL and 75 Vol% 23.1±2.3 μL/mL. At 4 hours the concentration of argon 25 Vol% was 16±1.6 μL/mL and significantly lower than argon 50 Vol% 26.9±1.2 μL/mL and argon 75 Vol% 27.2±0.2 μL/mL. There were neither statistically significant difference between argon concentrations after 2 or 4 hours of exposure nor between 50 and 75 Vol% argon.

**Table 1:** Argon concentration in cell culture medium.

	2 hours fumigation	4 hours fumigation
**Argon 75 Vol%**	**23.1±2.4**	**27.2±0.2**
**Argon 50 Vol%**	**24.9±1.4**	**26.9±1.2**
**Argon 25 Vol%**	**19.1±3.2** **	**16±1.6** ***

Concentration in medium referencing different concentrations of argon with 2 and 4 hours fumigation length respectively. Numbers are μL argon dissolved in mL cell culture medium (mean ± standard deviation of n = 8 individual experiments

** = p<0.01 and

*** = p<0.001).

### Argon treatment attenuates rotenone-induced apoptosis dose- but not time-dependently

**Fig 1:**
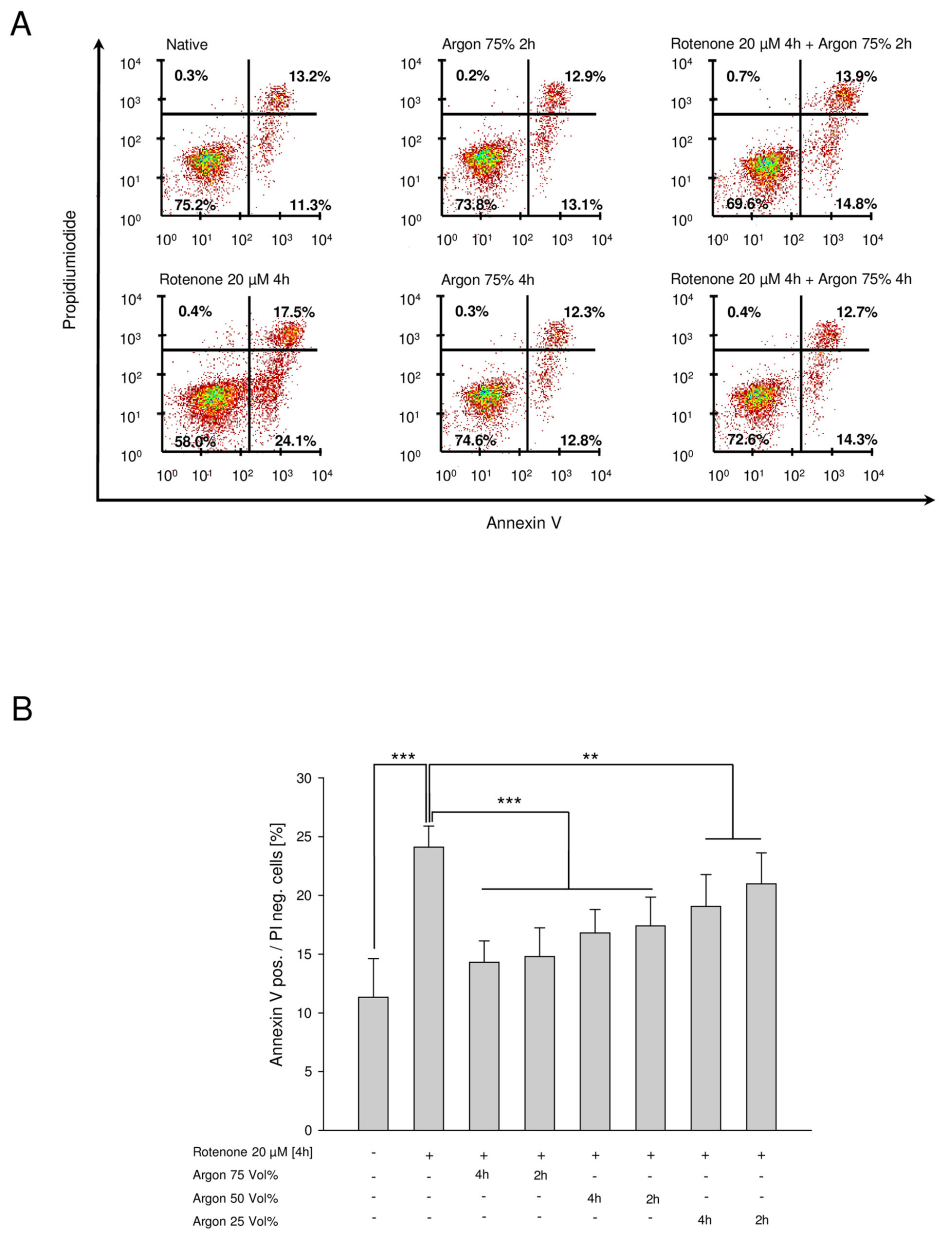
Argon attenuates rotenone-induced apoptosis dose-dependently but irrespective to length of treatment. **(A)** Representative dot-plots of the named intervention. **(B)** Annexin V positive and PI negative cells [%] of rotenone-induced apoptosis and consecutive argon treatment (n = 8; mean±SD; untreated 11.3±3.3% vs. rotenone 24.1±1.8% and rotenone 24.1±1.8% vs. argon 75 Vol% 14.3±1.8% and 50 Vol% 16.8±2.0% at 4 hours and rotenone 24.1±1.8% vs. argon 75 Vol% 14.8±2.4% and 50 Vol% 17.4±2.4% at 2 hours; all *** = p<0.001 and rotenone 24.1±1.8% vs. argon 25 Vol% 19.1±2.7% at 4 hours and rotenone 24.1±1.8% vs. argon 25 Vol% 21.0±2.7% at 2 hours; both ** = p<0.01).

Exposure of the cells to rotenone (20 μM for 4 h) increased the percentage of AV-positive and PI-negative cells from 11.3±3.3% to 24.1±1.8% (Fig 1B, Col. 1 and 2). Immediate treatment with argon inhibited the rotenone-induced increase in a dose dependent manner: Argon 75 Vol% exhibited a maximum effect and abolished rotenone treatment almost completely (Fig 1B, Col.2 and 3). Argon 50 Vol% showed a similar protective impact however to a reduced but still significant extent (Fig 1, Col. 2 and 5). Argon treatment with a concentration of 25 Vol% revealed a reduced but still significant effect (Fig 1B, Col. 2 and 7).

Argon´s effect was not dependent on length of exposure: a reduction from 4 h to 2 h exposure did not change the results concerning apoptosis for all three argon concentrations (Fig 1B, Col 4, 6 and 8). For all following experiments, we used argon 75 Vol% for 2 h to achieve the maximum effects.

### Argon suppresses rotenone-induced TLR2- and TLR4 cell surface receptor density and TLR2- and TLR4 protein expression

**Fig 2:**
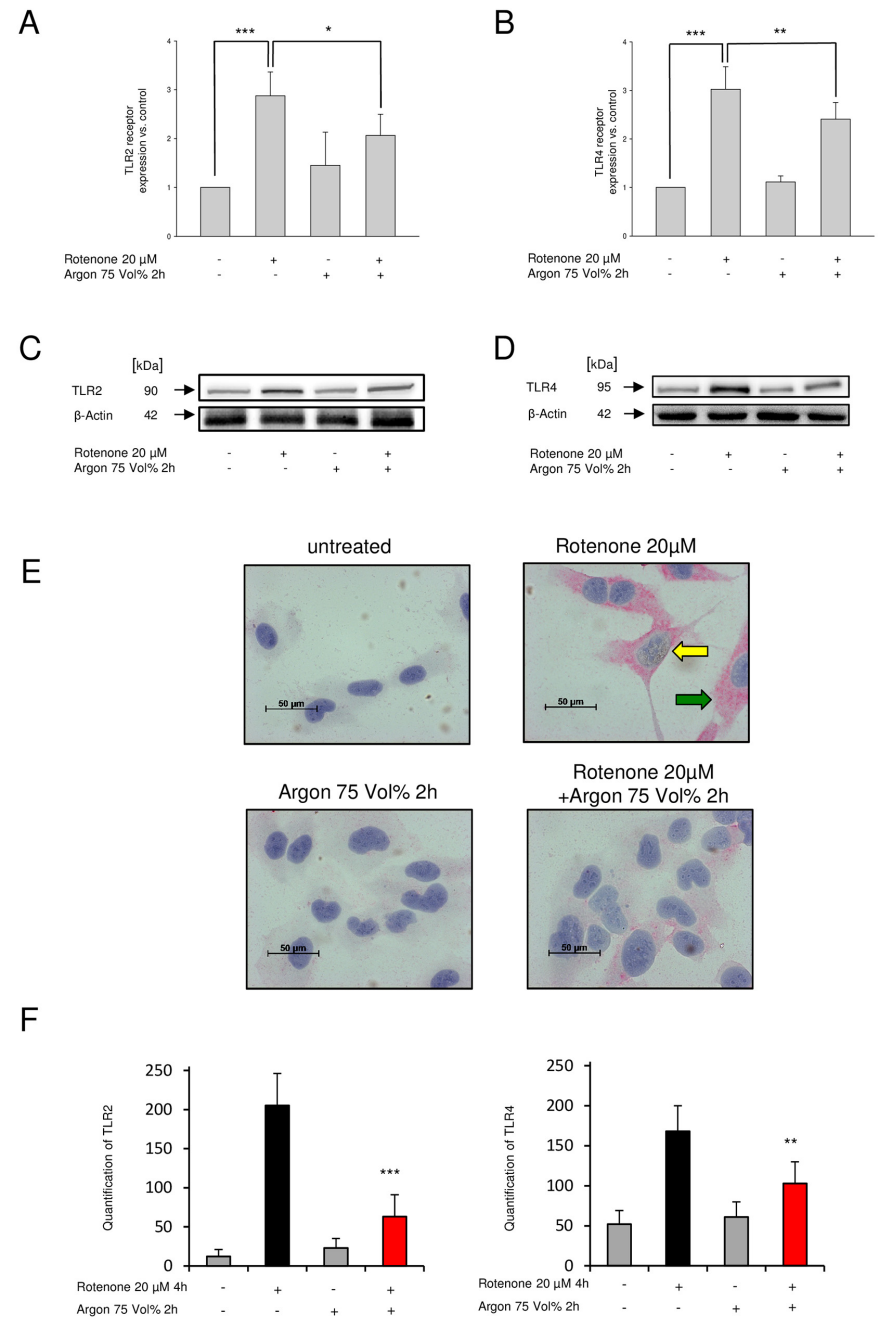
Argon reduces TLR2 and TLR4 receptor density. **(A and B)** FACS analysis of TLR2 and TLR4 receptor density on the cells´ surface (n = 8; mean±SD; rotenone 2.87±0.49% vs. rotenone+argon 75 Vol% [2 h] 2.070.4±3; * = p<0.05; rotenone 3.0±20.34 vs. rotenone+argon Vol%75 [2 h] 2.41±0.34; ** = p<0.01 and untreated was set to 1 vs. rotenone 2.87±0.49% and 3.02±0.46%; both *** = p<0.001). **TLR2 and TLR4 protein expression. (C and D)** Representing Western blot images, representing TLR2 (C) and TLR4 (D) protein expression after treatment with rotenone and argon (75 Vol%, 2 h). **TLR2 and TLR4 expression following argon treatment. (E)** Representative histological images of TLR2 (brown color) and TLR4 (red color) in differently treated cells (yellow arrow: TLR2 expression after rotenone treatment, green arrow: TLR4 expression after rotenone treatment). **(F)** Analysis of histogram quantification, left panel: TLR2 histogram (n = 5; TLR2 untreated 12±9; argon 75 Vol% 52±12; rotenone 205±41 vs. rotenone+argon 75 Vol% [2 h] 63±28; *** = p<0.001) and right panel: TLR4 histogram (n = 5; TLR untreated; 52±17, argon 75 Vol% 61±19; rotenone 168±32 vs. rotenone+argon 75 Vol% [2 h] 103±27; *** = p<0.01).

FACS analysis of TLR4 receptor revealed a significantly increased density of TLR4 receptors on the cells surface due to rotenone treatment (Fig 2A). Argon treatment of rotenone-induced apoptosis demonstrated a significant reduction of TLR2 receptor density (Fig 2B). Exemplary Western blot images showed an increased TLR2 and TLR4 protein expression after rotenone treatment while attenuated after argon fumigation (Fig 2C and 2D).

### Argon treatment reduces TLR2 and TLR4 expression in immunohistochemistry

Visualization of TLR2 and TLR4 following rotenone showed an increase in TLR2 and TLR4 expression. Following argon application, TLR2 and TLR4 expression vanished (Fig 2E). Quantification using histogram analysis revealed significance, regarding the argon-mediated reduction of TLR2 and TLR4 expression (Fig 2F).

### Argon mediated protective effects are attenuated by inhibition of TLR2- and TLR4-signaling

**Fig 3:**
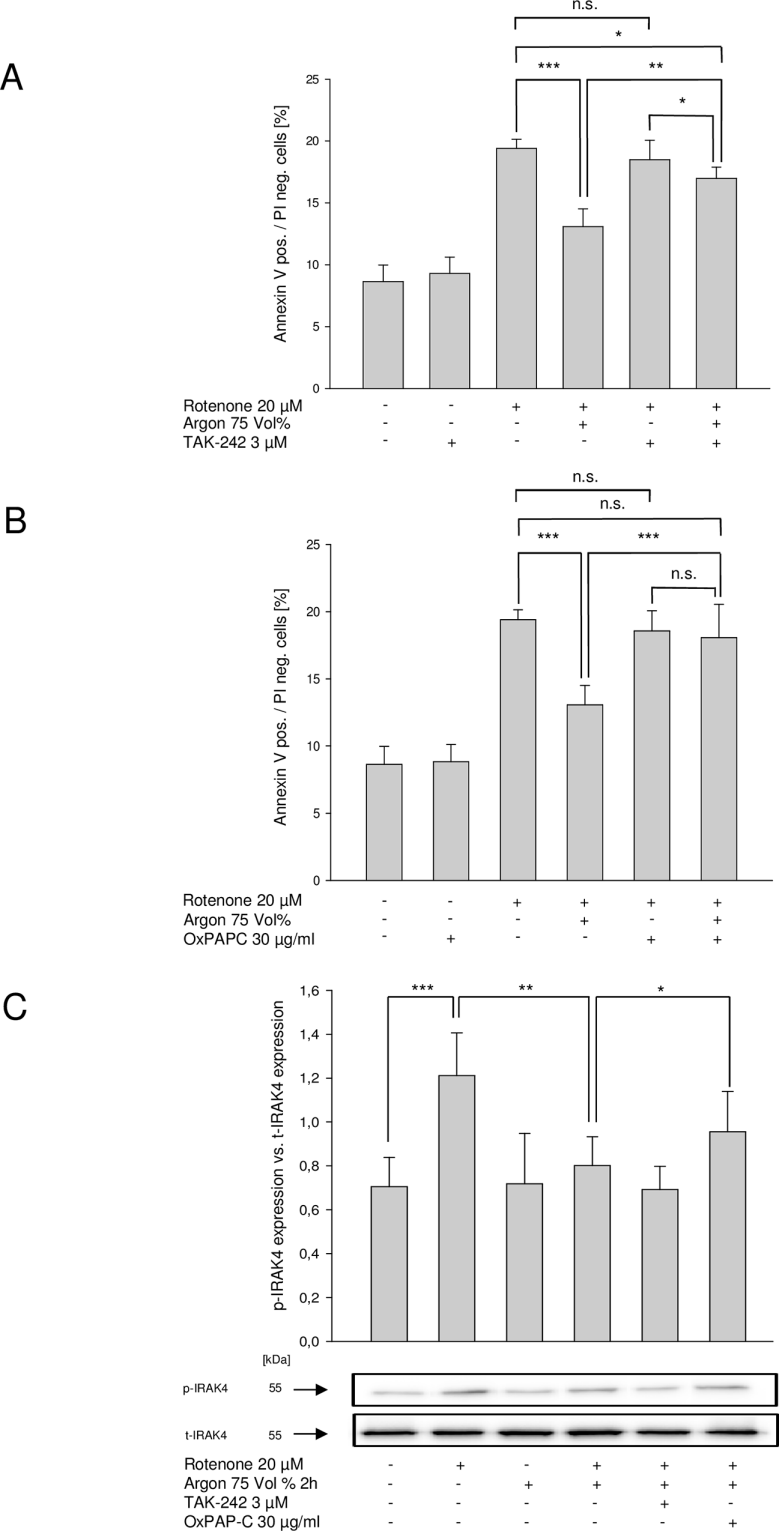
Argon-mediated cytoprotective effects are abolished after TLR2 and TLR4 inhibition. Annexin V positive and PI negative cells [%] of rotenone-induced apoptosis and consecutive argon treatment (n = 8; mean±SD; rotenone 19.4±0.7 vs. rotenone+argon 75 Vol% [2 h] 13.1±1.4; *** = p<0.001), **(A)** with TLR2 inhibitor (TAK-242; rotenone+argon 75 Vol% [2 h] 13.1±1.4 vs. TAK-242+rotenone+argon 75 Vol% [2 h] 16.9±1.0; ** = p<0.01 and rotenone 19.4±0.7 vs. TAK-242+rotenone+argon 75 Vol% [2 h] 16.9±1.0 and TAK-242+rotenone 18.5±1.6 vs. TAK-242+rotenone+argon 75 Vol% [2 h] 16.9±1.0; both * = p<0.05) or **(B**) TLR2 and TLR4 inhibitor (OxPAPC; rotenone+argon 75 Vol% [2 h] 13.1±1.4 vs. OxPAPC+rotenone+argon 75 Vol% [2 h] 18.1±2.9; *** = p<0.001, rotenone 19.4±0.7 vs. OxPAPC+rotenone+argon 75 Vol% [2 h] 18.1±2.9 and OxPAPC+rotenone 18.6±1.5 vs. OxPAPC+rotenone+argon 75 Vol% [2 h] 18.1±2.9; both n.s. = not significant). **Argon-mediated phosphorylation of IRAK4 is abolished after TLR2 and TLR4 inhibition. (C)** Densitometric analysis and representative Western blot image of IRAK4 protein phosphorylation after rotenone treatment. Equal loading was confirmed by reprobing with total-IRAK4 and analysis was performed in relation to the corresponding protein (n = 8; mean±SD; untreated 0.70±0.13 vs. rotenone 1.21±0.19; *** = p<0.001; rotenone 1.21±0.19 vs. rotenone+argon 75 Vol% [2 h] 0.80±0.13; ** = p<0.01 and rotenone+argon 75 Vol% [2 h] 0.80±0.13 vs. OxPAPC+rotenone+argon 75 Vol% [2 h] 0.92±0.2; * = p<0.05).

We incubated cells with TLR4 signaling inhibitor TAK-242 (3 μM for 60 min) and TLR2 and TLR4 signaling inhibitor OxPAPC (30 μg/ml for 60 min) alone and ascertained that there were no changes regarding apoptosis (Fig 3A and 3B, lanes 2). Next, we incubated cells with TAK-242 or OxPAPC before rotenone treatment, both showing no significant reduction of annexin V positive and PI negative cells (Fig 3A and 3B).

While the known effects of argon were reproducible, additional treatment with argon after TLR4 inhibition and rotenone still attenuated apoptosis and caused a significant decrease of AV-positive/PI-negative cells (Fig 3A). This effect of argon was completely abolished if TLR2 and TLR4 were both inhibited using OxPAPC. There was no significant reduction due to argon treatment anymore (Fig 3B).

### Argon inhibits IRAK4 but not MyD88 phosphorylation

Based on the previous findings, we next wanted to assess the influence of argon and the used inhibitors on proteins associated with the TLR downstream signaling pathway. SH-SY5Y cells treated with rotenone showed an increase of IRAK4 (Fig 3C). Argon treatment reduced rotenone-induced phosphorylation of IRAK4 significantly (Fig 3C, lane 4). OxPAPC but not TAK-242 significantly attenuated Argon mediated reduction of IRAK4 phosphorylation compared to rotenone and argon treatment (Fig 3C).

Although there was a tendency regarding a suppression of the phosphorylated form, western blot analysis of MyD88 in the context of rotenone and argon treatment did not reach significance (data not shown).

### Argon dependent increase in ERK1/2 phosphorylation is responsible for attenuated apoptosis

**Fig 4:**
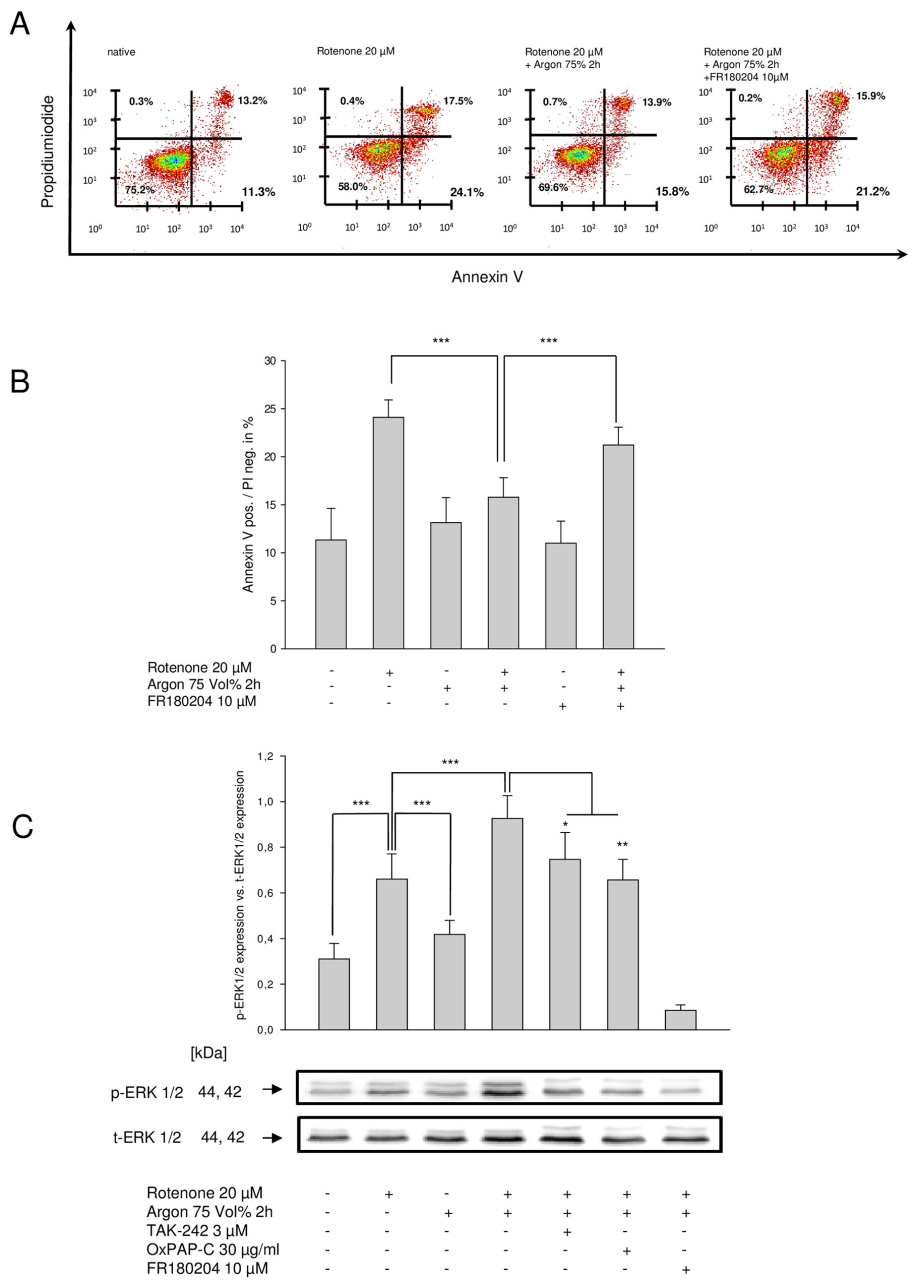
Argon´s cytoprotective properties are mediated via the phosphorylation of ERK1/2, which directly depends on TLR2 and TLR4 signalling. **(A)** Representative dot-plots of the particular intervention. **(B)** Annexin V positive and PI negative cells [%] of rotenone-induced apoptosis, consecutive argon treatment and inhibition of ERK1/2 using FR180204 (n = 8; mean±SD; rotenone 11.3±3.3% vs. rotenone+argon 75 Vol% [2 h] 13.1±2.6% and rotenone+argon 75 Vol% [2 h] 13.1±2.6% vs. FR180204+rotenone+argon 75 Vol% [2 h] 21.2±1.9%; both *** = p<0.001) **(C)** Densitometric analysis and representative Western blot image, demonstrating the phosphorylation of ERK1/2 protein expression after treatment with rotenone, argon and inhibitors of ERK, TLR2 and TLR4. Equal loading was confirmed by reprobing with the non-phosphorylated protein form (t-ERK; n = 8; mean±SD; untreated 0.31±0.07 vs. rotenone 0.66±0.11, rotenone 0.66±0.11 vs. argon 75 Vol% [2 h] 0.42±0.06 and rotenone 0.66±0.11 vs. rotenone+argon 75 Vol% [2 h] 0.93±0.10; all *** = p<0.001; rotenone+argon 75 Vol% [2 h] 0.93±0.10 vs. OxPAPC+rotenone+argon 75 Vol% [2 h] 0.66±0.09; ** = p<0.01 and rotenone+argon 75 Vol% [2 h] 0.93±0.10 vs. TAK-242+rotenone+argon 75 Vol% [2 h] 0.75±0.12, * = p<0.05).

While FR180204 alone had no effect, inhibition of ERK1/2 using FR180204 in combination with rotenone and argon, produced a significant increase of AV-positive/PI-negative cells compared to rotenone and argon treatment (Fig 4A and 4B).

Treatment with rotenone significantly induced ERK1/2 phosphorylation (Fig 4C). While argon treatment alone had no influence on ERK1/2 phosphorylation, argon treatment after rotenone increased ERK1/2 phosphorylation significantly and more than rotenone alone (Fig 4C). To further investigate this effect in the context of TLR inhibition, we evaluated ERK1/2 phosphorylation after exposure with TLR2 and TLR4 inhibitors. TLR4 inhibitor TAK-242 as well as TLR2 and TLR4 inhibitor OxPAPC attenuated argon-induced phosphorylation significantly (Fig 4C). Although a trend was detectable, no significant differences between the inhibitors could be measured.

### Argon treatment decreases rotenone-induced caspase-3 activity

**Fig 5:**
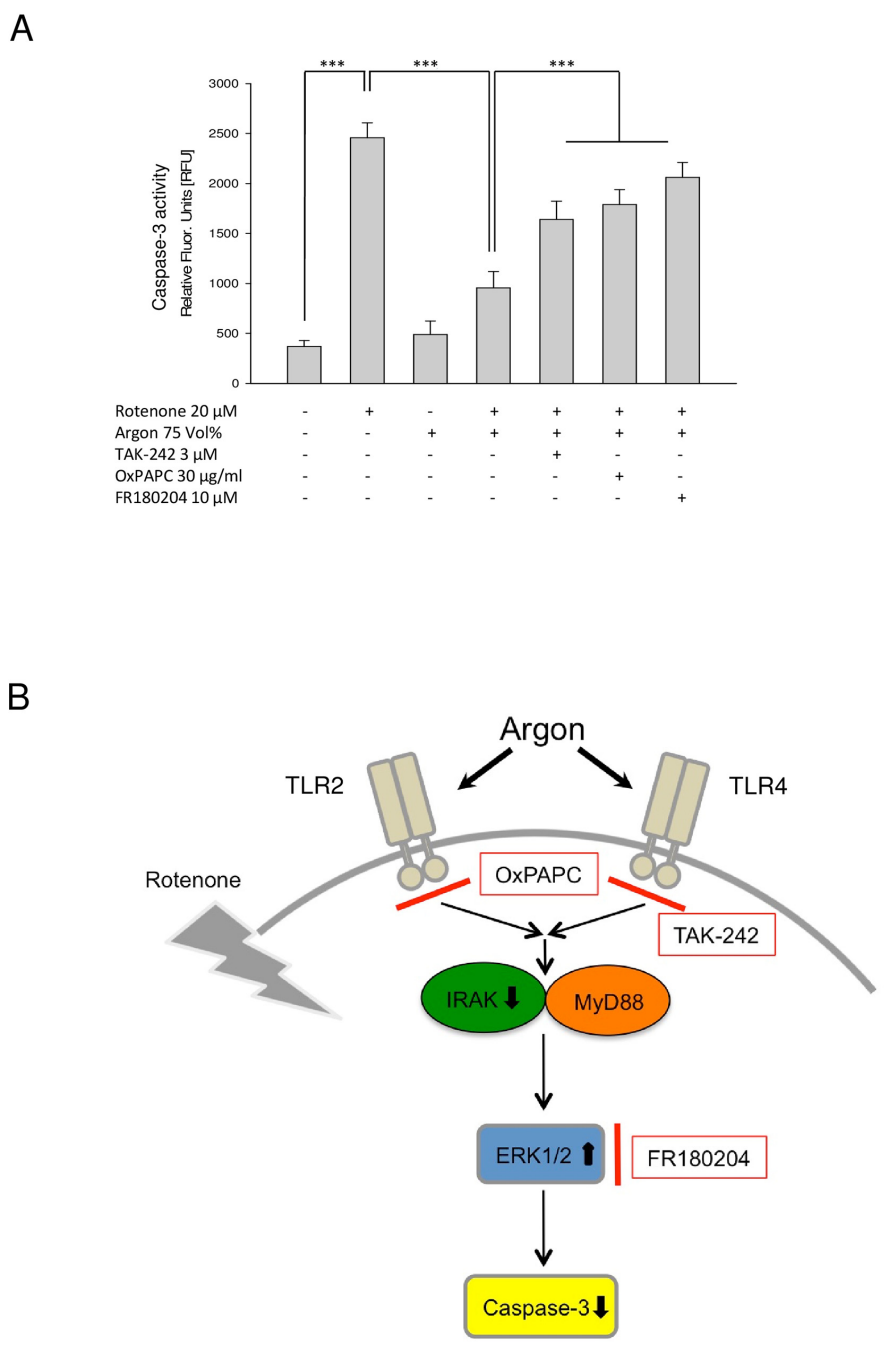
Argon-mediated decrease of apoptosis is attenuated following ERK1/2 and TLR inhibition. **(A)** Caspase activity assay demonstrating argon´s effect on enzyme activity in the context of different inhibitors (n = 8, mean±SD; untreated 369±60 vs. rotenone 2458±149; rotenone 2458±149 vs. rotenone+argon 75 Vol% [2 h] 956±163, rotenone+argon 75 Vol% [2 h] 956±163 vs. OxPAPC+rotenone+argon 75 Vol% [2 h] 1789±160, rotenone+argon 75 Vol% [2 h] 956±163 vs. TAK-242+rotenone+argon 75 Vol% [2 h] 1641±182 and rotenone+argon 75 Vol% [2 h] 956±163 vs. FR180204+rotenone+argon 75 Vol% [2 h] 2062±149; all *** = p<0.001). **(B) Diagram depicting the proposed mechanism of argon-mediated protective effects on neuronal cells.** Argon treatment affects both, TLR2 and TLR4, attenuates IRAK4 phosphorylation but not MyD88 and subsequently increases ERK1/2 phosphorylation in this model of rotenone-induced apoptosis. As a consequence, caspase-3 cleavage and activity are reduced thus conferring cytoprotection. Inhibition of TLRs and ERK1/2 MAPK abolished argon mediated effects regarding cytoprotection.

Rotenone significantly induced caspase-3 activity (Fig 5A). While argon alone had no effect on caspase-3 activity, argon treatment attenuated rotenone-induced caspase-3 activity significantly (Fig 5A, 4^th^ row). The inhibitors of TLR2, TLR4 and ERK1/2 (TAK-242, OxPAPC and FR180204) all antagonized argon-mediated inhibition of caspase-3 activity significantly. Although a trend was detectable, no significant differences between the inhibitors could be measured.

## Discussion

The main findings of this *in vitro* study can be summarized as follows: (1) Argon is soluble in cell culture medium and the partition equilibrium is reached in less than 2 h. (2) Argon exerts significant dose-dependent anti-apoptotic effect in human neuronal cells with argon 75 Vol% showing the most impressive effect. (3) Argon´s anti-apoptotic effects are not dependent on length of exposure. (4) Argon inhibited rotenone-induced apoptosis as shown by inhibition of AV-positive and PI-negative cells and caspase-3 activity. (5) Argon mediates anti-apoptotic signaling, decreasing TLR2 and TLR4 receptor density on the cells´ surface. (6) Argon attenuated IRAK phosphorylation but not MyD88 protein expression. (7) Argon increased ERK-1/2 phosphorylation. (8) Inhibition of TLR2, TLR4 and ERK1/2 attenuates argon-mediated anti-apoptotic effects. These results are discussed in detail:

To our best knowledge, no specifically *measured* concentrations of argon in aqueous solutions (cell culture media) have been published so far. Alderliesten and coworkers measured argon concentrations in blood of animals, and Auwärter et al. in post mortem blood and gas samples, but not in cell culture medium [14, 15]. Our gas chromatographic-mass spectrometric measurements of the argon concentrations revealed that 4 h of argon exposure did not lead to a further increase of concentrations when compared to 2 h exposure, thus suggesting that equilibrium was reached in less than 2 h. While argon 25 Vol% showed significantly lower mean concentrations for both exposure times (16 and 19.1 μL/mL), argon 50 and 75 Vol% resulted in mean argon concentrations in the range of 23.1 to 27.2 μL/mL.

We demonstrated that argon treatment attenuated rotenone-induced apoptosis in human neuronal cells mediating anti-apoptotic effects. Furthermore, the strongest effect was detected after immediate exposure with a concentration of 75 Vol% following argon 50% and 25%. Beyond that, even delayed argon exposure reduced rotenone-induced apoptosis. These results are in agreement with our previous *in vivo* findings showing the most effective concentration of argon at 75 Vol% while delayed application still showed significant protection [5]. In contrast, Loetscher *et al*. investigated different argon concentrations in their *in vitro* model of ischemic and traumatic brain injury and observed argon´s most protective effects are at a concentration of 50 Vol%, while 75 Vol% showed reduced but still significant protection [16]. In accordance with our findings, Bruecken *et al*. used argon at a dosage of 70 Vol% in a cardiac arrest model with rats and detected a protection with delayed argon treatment up to 3 hours after damage [17].

We demonstrated that the anti-apoptotic effect of argon was not significantly different after 2 h and 4 h treatment. However, there was a trend to further improvement due to longer exposure. This is in agreement with the argon concentrations measured in the cell culture medium. Bruecken *et al*. used an even shorter length of argon treatment (i.e. 1h) and demonstrated an improved neurological outcome after cardiac arrest [17]. These *in-vivo* results are in accordance with our previously published results, demonstrating argon´s effectiveness with inhalation for 1h [5, 6]. Further detailed dose- and time-dependent studies are necessary to determine the minimal time required for effective argon treatment.

Rotenone—a known inductor of apoptosis—was used in our *in vitro* model to initiate neuronal apoptosis [18, 19]. In accordance with our previous findings, we demonstrated that argon inhibits apoptotic signaling not only by inhibition of Bax, but by reduction of annexin-V positive / PI negative cells and an inhibition of caspase-3 activity [5]. Zhuang *et al*. confirm in their study that argon influences apoptotic signaling by increased synthesis of pro-survival proteins such as Bcl-2 [20]. Moreover, Spaggiari *et al*. were able to show that argon mediates cytoprotection and inhibits apoptosis. Argon treatment prevented mitochondrial membrane permeabilization, cytochrome c release, caspase-activation and avoided subsequent cell death [21]. In summary, argon´s anti-apoptotic effects seem partly to be mediated via the mitochondria and its special mechanisms.

Concerning the possible mechanism of argon-mediated cytoprotection, *in-vivo* experiments hypothesized a variety of potential target receptors on the cells surface, including the GABA-receptors as well as NMDA-receptors, potassium channels (TREK-1) or K_ATP_-channel blockers (i.e. 5-Hydroxydecanoate) which were all thought to be part the mechanisms. So far, no effects regarding cytoprotection could be measured at the mentioned receptors [7–10].

Although neurons are no typical immune cells, TLRs exert crucial function in neuronal cells. Toll-like receptors recognize molecules derived from microorganisms and activate immune cell response [22]. Previous studies revealed the presence of TLR2, TLR3, TLR4, and TLR8 in neurons [23, 24]. Monaghan *et al*. demonstrated that lipopolysaccharide (LPS) as part of the outer membrane of gram-negative cells, typically stimulating toll-like receptor 4, did not induce apoptosis in SH-SY5Y cells and hence concluded that these cells did not express TLR4 at all [25]. In contrast, Vaisid *et al*. affirmed in their study that both, TLR2 and TLR4 are present in human SH-SY5Y cells [26]. While the literature is discordant about this issue, TLR2 and TLR4 proteins and receptors were detectable in our present study.

Due to rotenone treatment, stressed cells release specific molecules and fragments stimulating toll-like receptors and activating toll-like receptor signaling [27–29]. Stimulated TLRs enable a pathway that leads among others to an interaction with the mitogen-activated protein kinase (MAPK) [30]. In view of these considerations we were able to demonstrate that rotenone significantly induces TLR2 and TLR4 receptor expression. Moreover, there was a significant reduction of TLR2 and TLR4 density on the surface of SY5Y cells due to argon treatment. FACS analysis makes it tempting to speculate, that argon may interact with the receptor, enabling its downstream targets to protect the cell. Stimulated TLRs are thought to recruit both the myeloid differentiation factor 88 (MyD88) and in combination with an interaction the interleukin-1 receptor associated kinase 4 (IRAK4) enhancing its phosphorylation. Thereafter, IRAKs dissociate and activate TAK1. This results in interaction with the mitogen-activated protein kinase (MAPK), especially ERK-1/2 [30]. In conclusion, we were able to demonstrate that rotenone induces IRAK4 but not MyD88 protein phosphorylation, while argon attenuated IRAK4 phosphorylation. The reasons, why argon does not include MyD88 induction but IRAK4 phosphorylation in this pathway remain unclear so far.

Inhibiting both, TLR4 using TAK-242 and TLR2 and TLR4 using OxPAPC partly provide protective effects. Ali *et al*. have reported that inhibition of TLR4 by TAK-242 protects porcine aortic endothelial cells against LPS stimulated apoptosis [31]. Tan *et al*. demonstrated a decrease in inflammatory responses in pulmonary artery endothelial cells while inhibiting TLR2 and TLR4 with OxPAPC [32]. We demonstrated in our investigation that TAK-242 could not prevent rotenone-mediated apoptosis. However, additional argon led to a significant decrease. Similarly, OxPAPC did not influence rotenone induced apoptosis. In contrast to TAK-242, TLR2 and TLR4 inhibition by OxPAPC argon could not reduce rotenone mediated apoptosis, so that OxPAPC inhibited argon´s protective effect. Reversely, we conclude that argon provides beneficial properties on rotenone induced apoptosis in SH-SY5Y cells by an inhibition of both toll-like receptors, TLR2 and TLR4.

Argon treatment increases ERK1/2 phosphorylation. This result is in accordance with our previous *in-vivo* findings in which we observed an increased ERK1/2 phosphorylation by argon postconditioning in rats. Inhibition of ERK1/2 abolished argon´s protective effects [6]. Fahlenkamp *et al*. confirmed in their *in-vitro* study argon´s competence to increase ERK1/2 phosphorylation [33]. We demonstrated that FR180204 –a highly selective ERK1/2 inhibitor–attenuates argon´s protective properties. Additionally, the inhibition of TLR2 and even more of TLR2 and TLR4 significantly decreased argon´s mediated increase in ERK1/2 phosphorylation.

In conclusion, the results of our *in-vitro* study suggest a novel and–so far–unknown molecular mechanism by which argon mediates its anti-apoptotic and neuroprotective properties. These effects may, at least in part, be executed via the TLR2 and TLR4 pathway (Fig 5B). Argon affects TLR-downstream signaling, modifying (among others) the phosphorylation of ERK1/2. In future, it is necessary to demonstrate these results in an *in-vivo* study, further expanding our knowledge regarding argon-mediated neuroprotection.
